# MDS/MPN With *SF3B1* Mutation and Thrombocytosis but Without Ring Sideroblasts

**DOI:** 10.1002/ajh.70040

**Published:** 2025-08-22

**Authors:** Biswadip Hazarika, Barbara J. Bain

**Affiliations:** ^1^ Department of Haematology Batra Hospital and Medical Research Centre New Delhi India; ^2^ Department of Immunology and Inflammation, St Mary's Hospital Campus of Imperial College Faculty of Medicine, Centre for Haematology St Mary's Hospital London UK

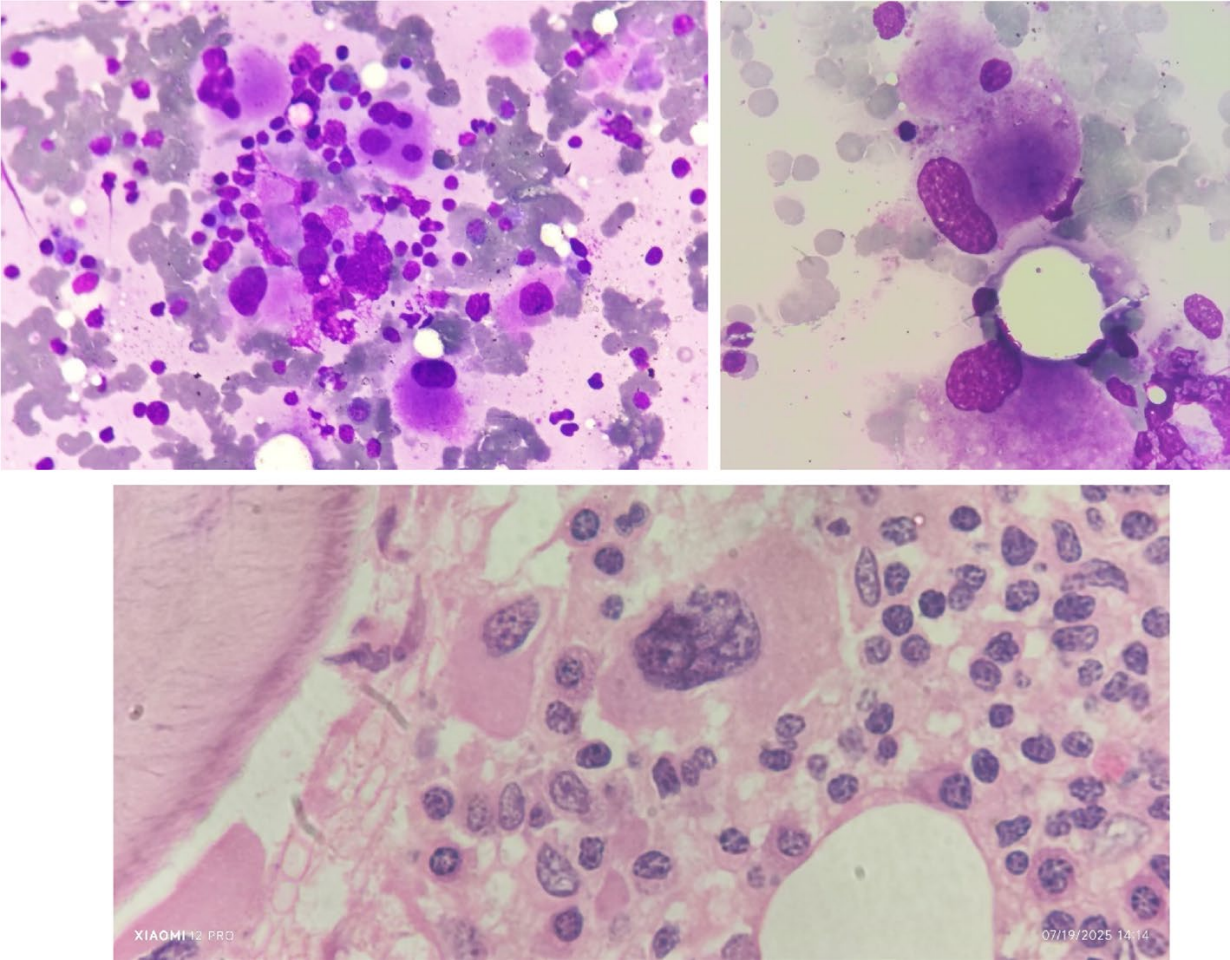

A 75‐year‐old man presented with extreme weakness and fatigability. On physical examination, there was no hepatomegaly or splenomegaly. He was found to be anemic with thrombocytosis. His blood count showed a white cell count of 3.46 × 10^9^/L, hemoglobin concentration 94 g/L, mean corpuscular volume 91.6 fL, mean corpuscular hemoglobin 28.1 pg., and platelet count 881 × 10^9^/L. Blood smear examination showed marked thrombocytosis and occasional hypochromic erythrocytes, but no other abnormalities. A bone marrow aspirate showed a mildly hypercellular marrow for the age of the patient with a myeloid: erythroid ratio of 3:1. Megakaryocytes were increased and of normal size with reduced lobulation (top left image ×40, top right ×100, May–Grünwald–Giemsa). Storage iron was increased (Grade 5/10) but, although siderotic granules were increased, a careful search disclosed no ring sideroblasts. Trephine biopsy sections showed hypercellularity with increased megakaryocytes, mostly with hypolobated or nonlobated nuclei (lower image, hematoxylin and eosin ×100) and Grade 2/4 reticulin fibrosis. His platelet count remained persistently elevated, between 850 and 1100 × 10^9^/L. Ultrasonography and computed tomography showed the spleen to be of normal size. Next‐generation sequencing (NGS) demonstrated a missense mutation in *SF3B1*, p.(Lys666Med) and frameshift insertions in *ASXL1*, p.(Gly646TrpfsTer12), and *STAG2*, p. (Glu750AsnfsTer2). Variant allele frequencies were 43%, 24%, and 30%, respectively. No mutation was detected in *BCR*, *ABL1*, *JAK2*, *CALR*, or *MPL*.

The disease features demonstrated did not meet the World Health Organization (WHO) criteria for myelodysplastic/myeloproliferative neoplasm (MDS/MPN) with *SF3B1* mutation and thrombocytosis as the presence of at least 15% ring sideroblasts is regarded as an essential diagnostic criterion [1]. Nor were the criteria for essential thrombocythemia met. Assignment is necessarily to the category MDS/MPN NOS (unclassifiable) [2]. In the International Consensus Classification (ICC), however, assignment to the category MDS/MPN with thrombocytosis and *SF3B1* mutation can be made as the presence of ring sideroblasts is regarded as “common but no longer required” for diagnosis [3]. With the move towards increasingly molecular criteria for classification of hematologic neoplasms the ICC criteria might be favored.

## Conflicts of Interest

The authors declare no conflicts of interest.

## Data Availability

No further data are available.
